# Modulation of glutaredoxin in the lung and sputum of cigarette smokers and chronic obstructive pulmonary disease

**DOI:** 10.1186/1465-9921-7-133

**Published:** 2006-10-25

**Authors:** Mirva J Peltoniemi, Paula H Rytilä, Terttu H Harju, Ylermi M Soini, Kaisa M Salmenkivi, Lloyd W Ruddock, Vuokko L Kinnula

**Affiliations:** 1 Biocenter Oulu and Department of Biochemistry, University of Oulu, Oulu, Finland; 2 Department of Internal Medicine, University of Oulu, Oulu, Finland; 3 Department of Medicine, Division of Allergology, University of Helsinki, Helsinki, Finland; 4 Department of Pathology, Oulu University Hospital, Oulu, Finland; 5 Department of Pathology, Helsinki University Hospital, Helsinki, Finland; 6 Biomedicum Helsinki and Department of Medicine, Division of Pulmonary Diseases, PO Box 340 (Haartmaninkatu 4), 00029 Helsinki University Hospital, Helsinki, Finland

## Abstract

**Background:**

One typical feature in chronic obstructive pulmonary disease (COPD) is the disturbance of the oxidant/antioxidant balance. Glutaredoxins (Grx) are thiol disulfide oxido-reductases with antioxidant capacity and catalytic functions closely associated with glutathione, the major small molecular weight antioxidant of human lung. However, the role of Grxs in smoking related diseases is unclear.

**Methods:**

Immunohistochemical and Western blot analyses were conducted with lung specimens (n = 45 and n = 32, respectively) and induced sputum (n = 50) of healthy non-smokers and smokers without COPD and at different stages of COPD.

**Results:**

Grx1 was expressed mainly in alveolar macrophages. The percentage of Grx1 positive macrophages was significantly lower in GOLD stage IV COPD than in healthy smokers (p = 0.021) and the level of Grx1 in total lung homogenate decreased both in stage I–II (p = 0.045) and stage IV COPD (p = 0.022). The percentage of Grx1 positive macrophages correlated with the lung function parameters (FEV1, r = 0.45, p = 0.008; FEV1%, r = 0.46, p = 0.007, FEV/FVC%, r = 0.55, p = 0.001). Grx1 could also be detected in sputum supernatants, the levels being increased in the supernatants from acute exacerbations of COPD compared to non-smokers (p = 0.013) and smokers (p = 0.051).

**Conclusion:**

The present cross-sectional study showed that Grx1 was expressed mainly in alveolar macrophages, the levels being decreased in COPD patients. In addition, the results also demonstrated the presence of Grx1 in extracellular fluids including sputum supernatants. Overall, the present study suggests that Grx1 is a potential redox modulatory protein regulating the intracellular as well as extracellular homeostasis of glutathionylated proteins and GSH in human lung.

## Background

The pathogenesis of chronic obstructive pulmonary disease (COPD) is probably strongly associated with reactive oxygen metabolites. Cigarette smoke not only contains high levels of oxidants, but it also leads to the accumulation of neutrophils and macrophages in the lung and to their activation [1-3]. A number of studies have investigated antioxidant defense mechanisms in cigarette smoke exposed cells and in chronic cigarette smokers. These studies have found that glutathione (GSH), a thiol containing tripeptide present in the epithelial lining fluid (ELF) at high concentrations, plays an essential role in protecting human airways against exogenous and endogenous oxidants and cigarette smoke [1,4,5]. However, only some of the enzyme systems participating in GSH regulation, and thereby probably also participating in COPD pathogenesis, have been investigated in human lung.

Glutathione is present in increased concentrations in the ELF of chronic smokers [6] and both acute and chronic exposure of experimental animals to cigarette smoke causes depletion in the intracellular GSH concentration [7]. GSH is transported from cells by multiple mechanisms, while the plasma membrane is impermeable to GSH preventing its transportation back into cells. The replenishment of intracellular GSH is accomplished by the reduction of oxidized glutathione, i.e. glutathione disulphide (GSSG), release of GSH from the proteins and de novo GSH synthesis [8]. Enzyme mechanisms that are known to regulate GSH metabolism include the rate-limiting enzyme in GSH synthesis, glutamate cysteine ligase (GCL, also known as γ-glutamylcysteine synthetase, γ-GCS), glutathione peroxidases (GPx), glutathione reductase (GR), γ-glutamyltranspeptidase (γ-GT) and glutathione-S-transferases (GST). There appears to be increased mRNA expression of GCL, GPxs and some GSTs in the bronchial epithelium of chronic smokers, but decreased immunoreactivities or activities of several of these enzymes in cigarette smokers or during COPD progression [9]. Glutaredoxins (Grx) represent a redox modulatory protein family with potential effects on GSH regulation and homeostasis, but until now they have not been assessed in smoking related lung diseases.

Classical glutaredoxins are small thiol disulfide oxidoreductases with a conserved active site sequence -*CXXC*- and a GSH binding site. They belong to the thioredoxin fold superfamily [10,11], thioredoxin being a known redox modulatory enzyme in human lung [12,13]. There are two Grxs in humans, cytosolic Grx1 and mitochondrial Grx2 [14,15]. They catalyze disulfide reductions, preferring GSH-mixed disulfides as substrates, by utilizing the reducing power of GSH in the presence of NADPH and glutathione reductase [16]. Grxs can be hypothesized to participate in the reduction of the GSH-mixed disulfides of thiol-containing proteins back to their active forms during and after oxidative stress in cigarette smokers and in COPD.

In this study the expression of Grxs was investigated in lung specimens of non-smokers and cigarette smokers and in different stages [17] of COPD or emphysema/COPD associated with α-1-antitrypsin deficiency (GOLD stages I, II and IV). The major focus was on macrophages, as Grx1 is mainly expressed in these cells [18] and since one typical feature in COPD is the accumulation of macrophages in the lung. Given that Grx1 may be present also in the extracellular space [19,20], levels of Grx1 were also examined from induced sputum specimens, both cells and supernatants obtained from non-smokers, smokers and COPD patients.

## Materials and methods

### Tissues

The tissue material included uninvolved peripheral lung tissue from lung surgery (hamartomas, carcinoid tumors, lung carcinomas) representing healthy lung from non-smokers, smokers without COPD and stage I–II COPD (Department of Pathology, Oulu University Hospital). The specimens from patients with very severe COPD or emphysema from α-1-antitrypsin deficiency (stage IV) were retrieved from lung transplantations (Department of Pathology, Helsinki University Hospital). The tissue material for immunohistochemistry was preserved as described by Dail and Hammar [21]. More detailed description can be found from the accompanying data supplement [see Additional file 1]. The tissue material for the Western analysis was immediately frozen in liquid nitrogen and stored in -80°C. Patient characteristics for specimens containing separate subjects used in immunohistochemistry (n = 44) and in Western blotting (n = 32) are shown in Table 1 and Table 2, respectively.

**Table 1:** Clinical characteristics of the patients in immunohistochemistry analyses

	**Non-smokers**	**Smokers**	**COPD st I–II**	**COPD st IV**	**p-value**
**Total (n)**	9	10	10	16	
**Male/Female**	2/7	9/1	7/3	10/6	
**Age (years)**	66 (13)	62 (7)	60 (8)	54 (7)	
**Pack years**	0	51 (11)	32 (18)	29 (17)	p < 0.0001
**FEV1 (l)**	2.7 (1.4)	3.0 (0.8)	2.4 (0.8)	0.6 (0.3)	p < 0.0001
**FEV1 (% of ref)**	104 (16)	85 (10)	73 (13)	20 (10)	p < 0.0001
**FEV1/FVC (%)**	85 (10)	79 (7)	70 (12)	34 (9)	p < 0.0001
**DCO (% of ref)**	91 (18)	79 (15)	69 (13)	25 (8)	p < 0.0001
**DCO/VA (% of ref)**	90 (12)	83 (12)	71 (14)	37 (10)	p < 0.0001

Data is shown as mean (SD), as % of reference where applicable. None of the patients with stage I–II disease were on oral or inhaled corticosteroids. All patients with stage IV COPD had inhaled corticosteroid (below 1 mg). None of the patients were on N-acetylcysteine or vitamins. No one had previous asbestos exposure.

**Table 2:** Clinical characteristics of the patients in Western blot analyses

	**Non-smokers**	**Smokers**	**COPD st I–II**	**COPD st IV**	**p-value**
**Total (n)**	8	9	9	6	
**Male/Female**	3/4*	6/3	6/3	5/1	
**Age (years)**	64 (12)	60 (7)	64 (9)	53 (6)	
**Pack years**	0	36 (15)	37 (12)	32 (22)	p < 0.0001
**FEV1 (l)**	2.9 (1.1)	3.0 (0.6)	2.0 (0.5)	0.5 (0.2)	p < 0.0001
**FEV1 (% of ref)**	99 (21)	87 (15)	68 (14)	16 (7)	p < 0.0001
**FEV1/FVC (%)**	88 (6)	82 (11)	73 (12)	35 (12)	p < 0.0001
**DCO (% of ref)**	92 (20)	83 (8)	65 (14)	29 (8)	p < 0.0001
**DCO/VA (% of ref)**	95 (19)	88 (8)	71 (17)	38 (10)	p < 0.0001

Data is shown as mean (SD), as % of reference where applicable. *One healthy organ donor, background details missing. Three patients with stage II COPD and all patients with stage IV COPD had inhaled corticosteroid (below 1 mg). None of the patients were on N-acetylcysteine or vitamins. No one had previous asbestos exposure.

### Sputum induction and processing

A standard procedure for sputum induction was conducted using 4.5% hypertonic saline given at 5-minute intervals for a maximum of 20 minutes according to the guidelines of the European Respiratory Society's Task Force [22]. Details are described in the data supplement [see Additional file 1]. The sputum specimens were immediately centrifuged, frozen and stored at -80°C. The characteristics of the patients selected for studies on induced sputum specimens (n = 50) are shown in Table 3. The subjects included non-smokers, healthy smokers and mild stable COPD (stage 0–I) and, for comparison, cases with more severe disease and hospitalization due to COPD exacerbations (COPD exa). The patients did not suffer from other diseases or pneumonia. All samples from patients with COPD exacerbation were collected within 48 hours of admission. Symptoms were assessed with the St George's Respiratory Questionnaire.

**Table 3:** Clinical information of the patients included in induced sputum experiments

	**Non-smokers**	**Smokers**	**stable COPD**	**COPD exa**	**p-value**
**Total (n)**	15	11	17	7	
**Male/Female**	11/4	9/2	11/6	5/2	
**Age (years)**	57 (6)	51 (6)	61 (8)	61 (7)	
**Pack years**	0	31 (10)	40 (15)	35 (7)	p < 0.0001
**FEV1 (l)**	3.6 (0.6)	3.9 (0.9)	2.2 (0.8)	1.1 (0.7)	p < 0.0001
**FEV1 (% of ref)**	101 (10)	98 (11)	67 (20)	35 (20)	p < 0.0001
**FEV1/FVC (%)**	81 (5)	81 (4)	66 (12)	47 (9)	p < 0.0001
**DCO (% of ref)**	97 (9)	87 (12)	64 (16)	36 (22)	p < 0.0001
**DCO/VA (% of ref)**	105 (10)	94 (9)	75 (21)	48 (28)	p < 0.0001

Data is shown as mean (SD), as % of reference where applicable. None of the patients with stable disease were on oral or inhaled corticosteroids. The stable COPD represented patients with mild stage 0–I COPD and COPD exa group represented patients with COPD exacerbation as described. Oral corticosteroid and antibiotic had been started to all patients with COPD exacerbation within the last 48 hours. None of the patients were on N-acetylcysteine or vitamins.

### Immunohistochemistry and assessment of Grx1 in the lung

The preparation and staining protocol for paraffin sections using polyclonal goat anti-human Grx1 antibody [19] has been described earlier [18,23]. More detailed description is provided in the data supplement [see Additional file 1]. The immunohistochemical staining intensities were assessed semiquantitatively from ten microscopic fields by two investigators by grading the staining intensity from negative (0) to strong (3). In addition, at least ten microscopic fields were evaluated by calculating the absolute numbers of Grx1 positive and negative macrophages with the Zeiss AxioHOME Morphometry program (Zeiss, Jena, Germany).

### Western blot analyses

Frozen tissue samples were quickly homogenized in ice cold phosphate buffered saline. Western blot analyses from tissue homogenates and sputum specimens were conducted as described earlier [18,23] with antibodies against Grx1 [19] and Grx2 [15]. A more detailed description of the method can be found in the data supplement [see Additional file 1]. β-Actin was not used as a loading control for tissue homogenates since it showed high individual variability as has been shown earlier [24,25]. Instead, the protein concentration was measured carefully as triplicates from a set of samples and equal loading was ensured by staining the blotted membranes with Ponceau S (Sigma Aldrich, St. Louis, MO, USA).

### Ethical considerations

The study protocol was accepted by the ethical committees of Oulu University Hospital and Helsinki University Hospital and it is in accordance with the ethical standards of the Helsinki declaration of 1975. The study was registered by the Helsinki University Hospital [26].

### Statistics

Statistical analyses were performed with the Statistical Package for Social Studies (SPSS) version 11.5 (Chicago, IL, USA). Differences between controls and selected diseases were compared using analysis of variance and post hoc comparisons were performed using two-tailed *t *tests. Categorical data were compared using Fisher's exact test. *P*-values less than 0.05 were considered statistically significant. Correlations to lung functions were analyzed with the Spearman rank correlation test. The two independent evaluations of immunohistochemical samples were compared using Cohen's kappa statistics [27].

## Results

### The number of alveolar macrophages in tissue specimens

**Figure 1:**
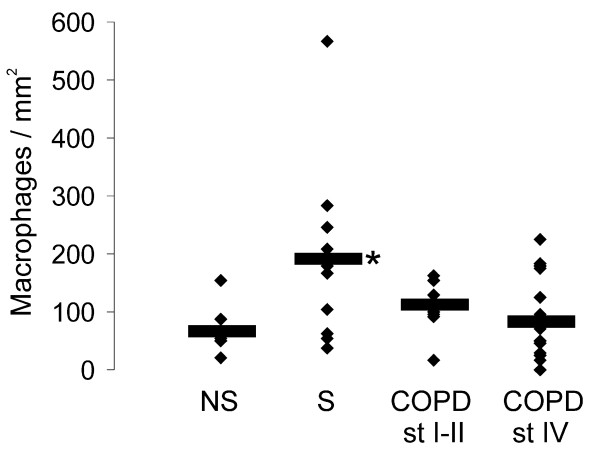
**Number of alveolar macrophages**. The number of alveolar macrophages was assessed in healthy non-smokers (NS), smokers without COPD (S), stage I–II COPD and stage IV COPD (for patient characteristics see Table 1). The means of the numbers of alveolar macrophages per square millimeter are marked with horizontal lines. * Significantly increased when compared to non-smokers (p = 0.020).

Since Grx1 is known to be highly expressed in alveolar macrophages [18], a detailed analysis was conducted where the number of alveolar macrophages per square millimeter of tissue specimen was analyzed from all groups of subjects i.e. non-smokers, smokers, stage I–II COPD and stage IV COPD. These analyses showed an increased number of macrophages especially, in the lung tissue of smokers (p = 0.020) compared to non-smokers (Fig 1).

### Grx1 in healthy human lung and in different stages of COPD

**Figure 2:**
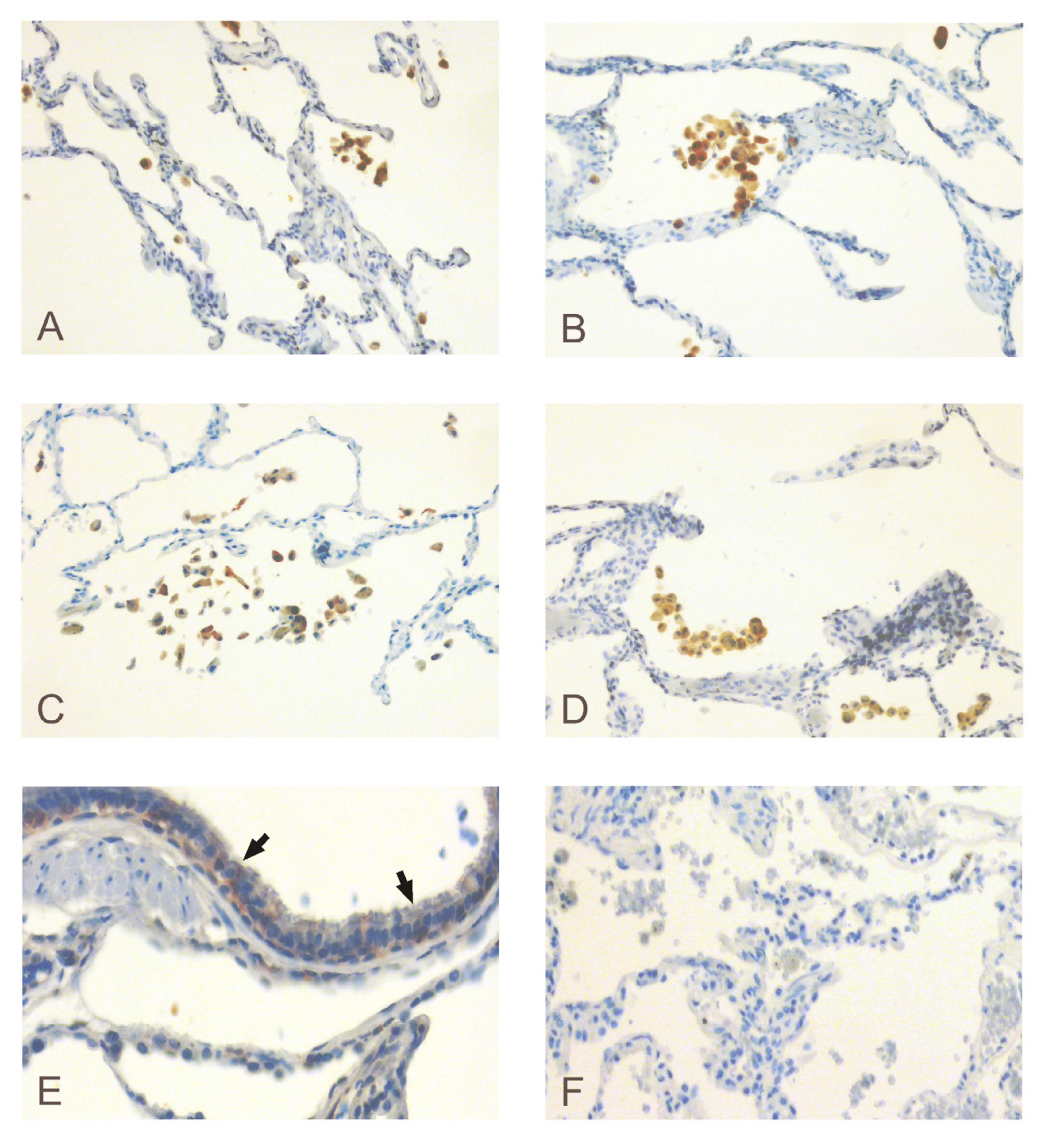
**Immunohistochemical expression of Grx1 in lung specimens**. Representative immunohistochemical expression of Grx1 in lung specimens from a healthy non-smoker (A), smoker without COPD (B), stage II COPD (C) and stage IV COPD (D). Grx1 was mainly expressed in alveolar macrophages in all cases. Occasional positivity was observed in the bronchial epithelium as indicated by arrows (E). No immunoreactivity was seen in the negative isotype control (F). Dilutions used were 1:3000 for goat anti-human Grx1 primary antibody and 1:300 for biotinylated rabbit anti-goat secondary antibody.

Immunohistochemical studies on healthy lung and COPD showed that Grx1 was mainly expressed in alveolar macrophages (Fig 2A–D). Occasional faint positivity was also detected in the bronchial epithelium in some cases (Fig 2E). No immunoreactivity was seen in the negative isotype control (Fig 2F). There were no statistically significant differences (p = 0.097) in the semiquantitative analysis of the immunohistochemical staining intensities between any two groups of subjects. The evaluations by two independent investigators showed moderate agreement with each other according to Cohen's kappa statistics (κ = 0.552).

**Figure 3:**
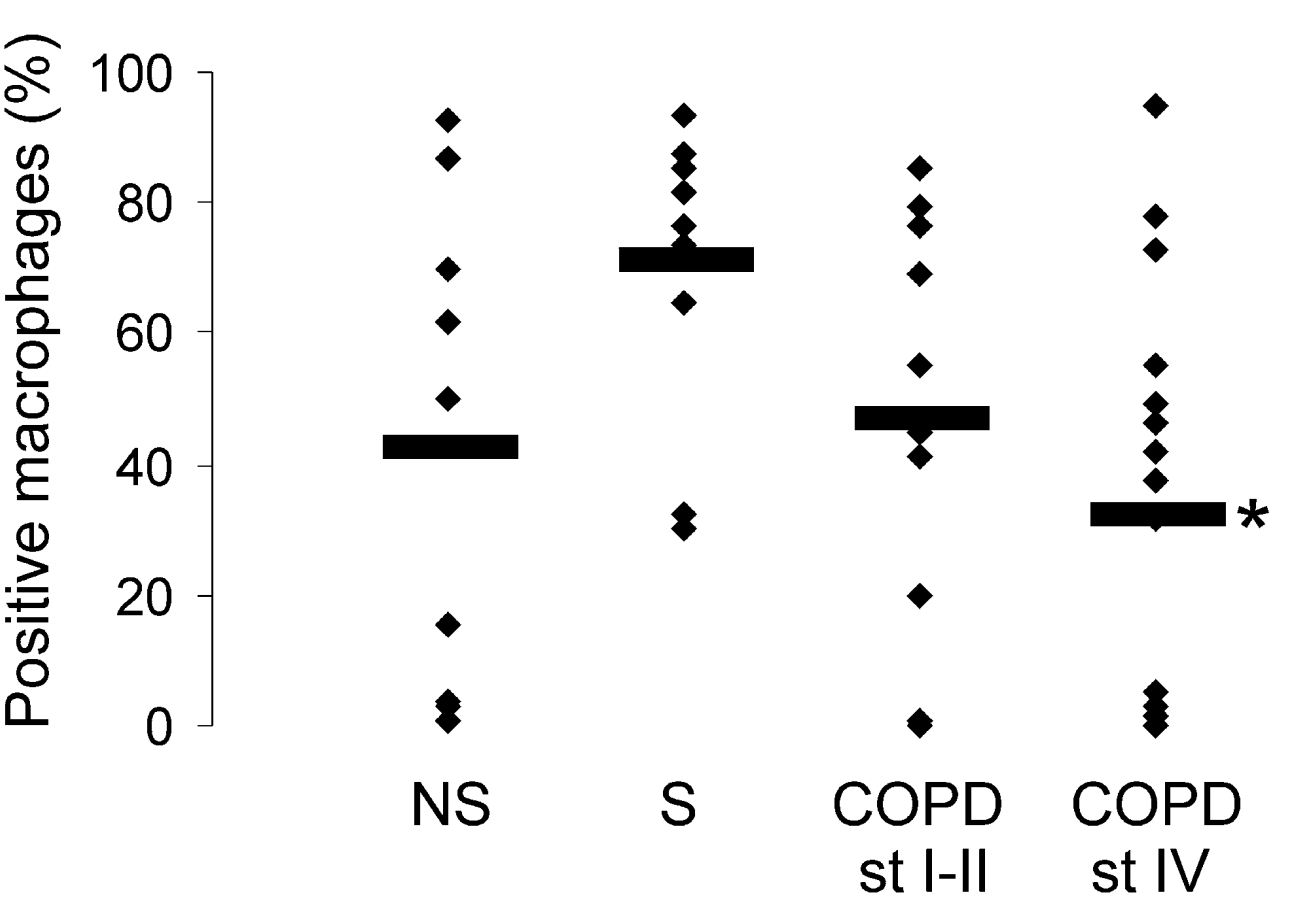
**Analysis of Grx1 positive macrophages**. The percentage (%) of Grx1 positive macrophages from the total number of alveolar macrophages in healthy non-smokers (NS), smokers without COPD (S), stage I–II COPD and stage IV COPD (for patient characteristics see Table 1). The means of the percentage of positive macrophages are marked with horizontal lines. * Significantly decreased when compared smokers (p = 0.021).

**Figure 4:**
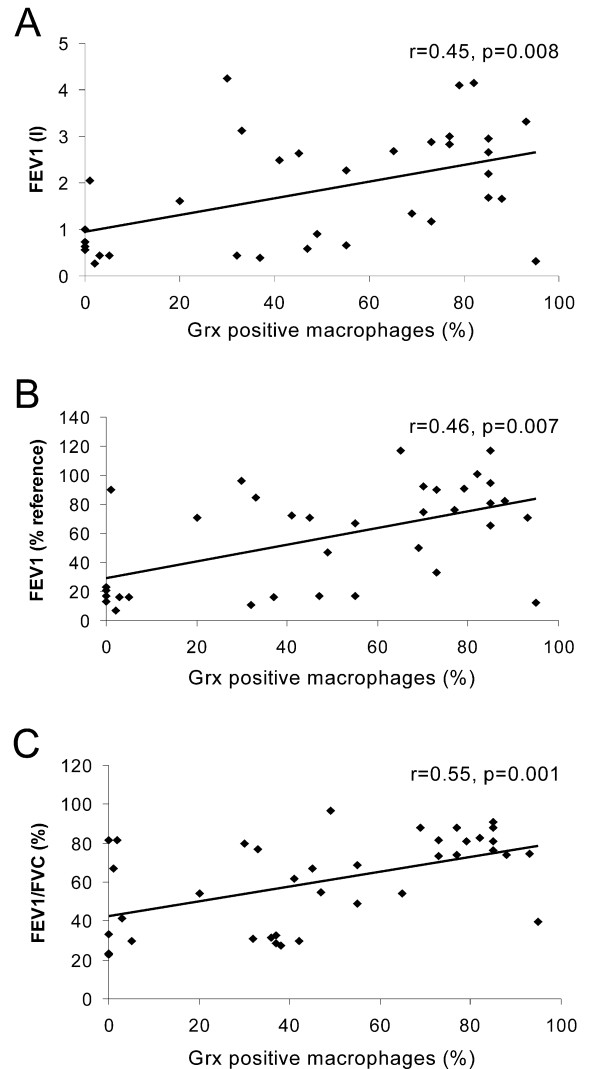
**Correlation of tissue Grx1 with lung function parameters**. Correlations between the percentage of Grx1 positive macrophages (of total macrophage population examined) and lung function parameters. The percentage of Grx1 positive macrophages was observed to significantly correlate with FEV1 (r = 0.45, p = 0.008), FEV1% (r = 0.46, p = 0.007) and FEV/FVC% (r = 0.55, p = 0.001).

When the cells were divided into two groups, either Grx1 positive or negative, the percentage of Grx1 positive macrophages from the total macrophage population showed a tendency to decrease during disease progression, being lowest in stage IV COPD. This reduction was statistically significant when compared to smokers (p = 0.021) (Fig 3). There was no association between the pack years and the percentage of positive macrophages. Interestingly, there was a significant correlation between Grx1 positive macrophages in the lung tissue specimens (containing lung samples from all smokers and different stages of COPD) and lung function parameters (FEV1, r = 0.45, p = 0.008; FEV1%, r = 0.46, p = 0.007, FEV/FVC%, r = 0.55, p = 0.001) as shown in figure 4 (panels A-C).

**Figure 5:**
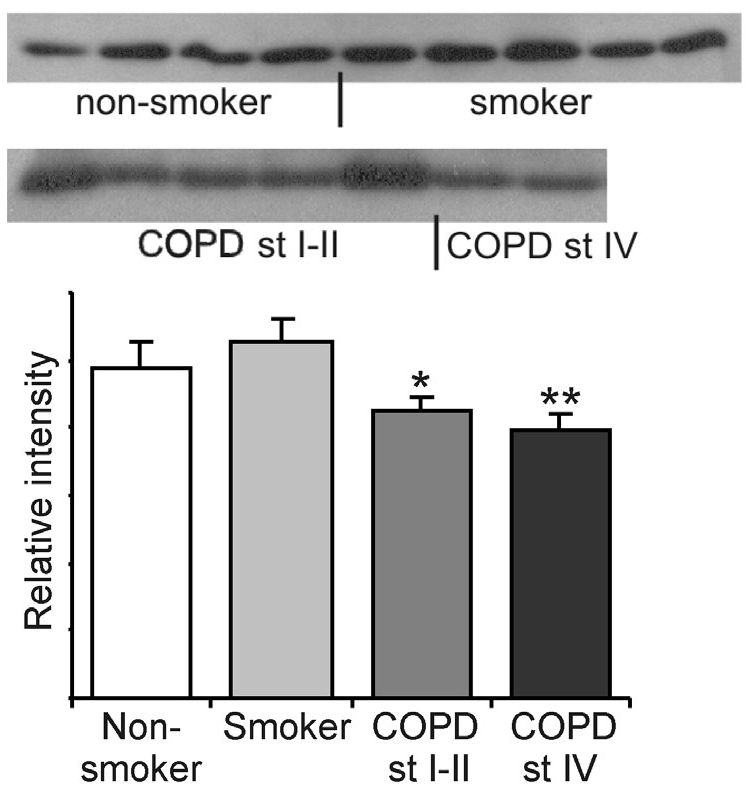
**Western blot analysis of Grx1 in lung homogenates**. Representative Western blot analysis of Grx1 expression in the specimens (n = 32) of healthy non-smokers and smokers without COPD and in different stages of COPD (for patient characteristics see Table 2). The total amount of protein was 40 μg and the primary antibody dilution for human Grx1 was 1:2500 in all cases. The means of the intensities measured are shown as columns with error bars representing SEM. * Significantly decreased when compared to smokers (p = 0.045). ** Significantly decreased when compared to smokers (p = 0.022).

In agreement with this result the Western blot analysis of lung homogenates (n = 32) showed that Grx1 levels were decreased in COPD when compared to healthy smokers (stage I–II p = 0.045 and stage IV p = 0.022) (Fig 5). In these subjects, there was no statistically significant difference in the pack years between smokers and COPD (p = 0.818).

Unlike cytosolic Grx1, mitochondrial Grx2 could not be detected by immunohistochemistry. In the Western blot analysis Grx2 was hardly detectable and was only found in cigarette smokers (data not shown).

### Grx1 in the induced sputum supernatants

**Figure 6:**
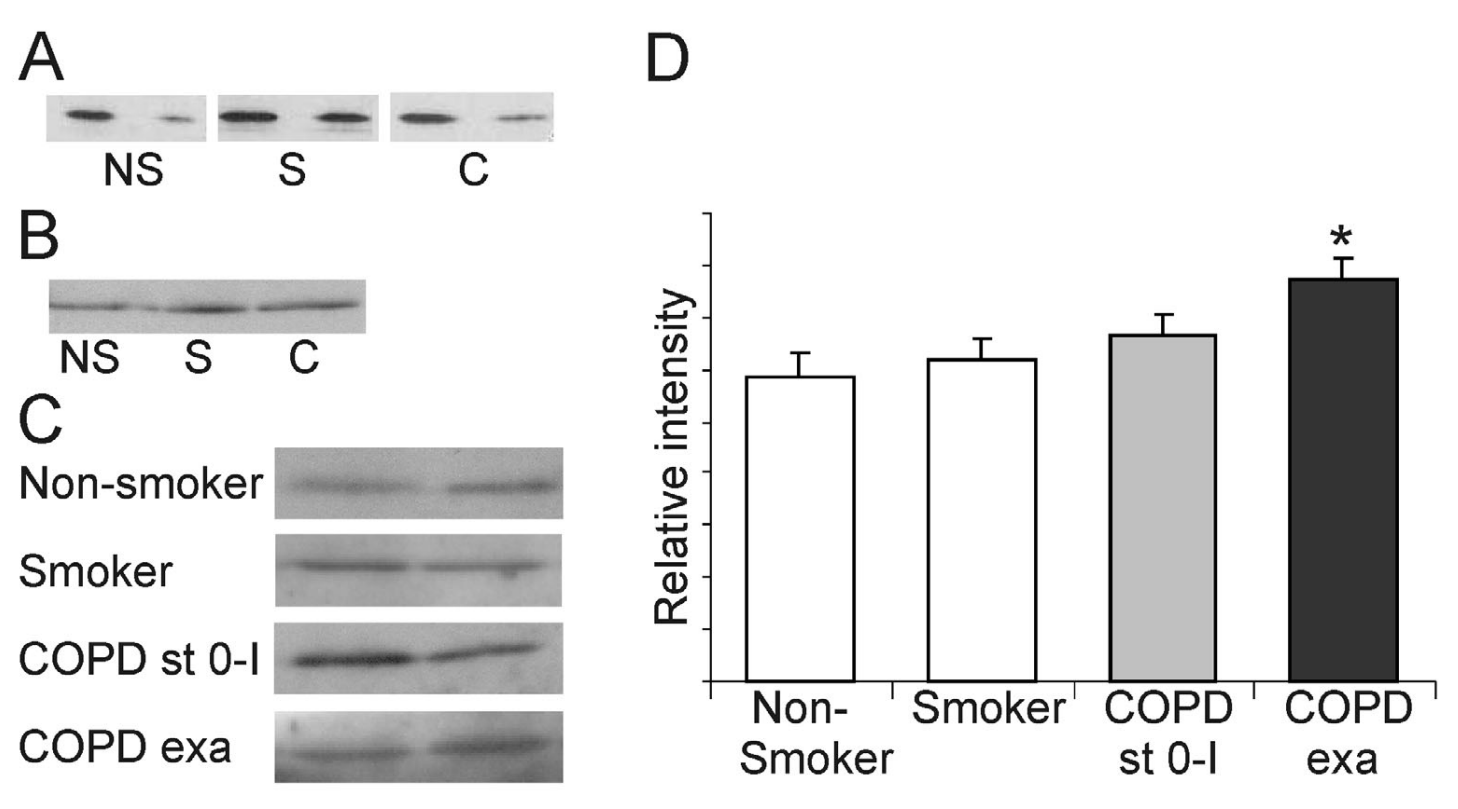
**Grx1 expression in induced sputum supernatants and plasma samples**. Representative Western blot analysis of Grx1 expression in the plasma (A) and induced sputum pellets (B) and supernatants (C) of healthy non-smokers (= NS), smokers (= S) and COPD patients (= C). The means of the intensities in the sputum supernatants were measured from total of 15 non-smokers, 11 smokers, 17 stable COPD (stage 0–I) and 7 acute exacerbations (for patient characteristics see Table 3) and are shown as columns (D). The primary antibody dilution for human Grx1 was 1:2500 in all cases. Error bars represent SEM. * Significantly increased when compared to non-smokers (p = 0.013).

Grx1 could be detected by Western blot analysis in the plasma samples from non-smokers, smokers and COPD patients, but with high individual variability (see also Fig 6A) confirming previous findings on Grx1 in plasma [19,20]. Since the intracellular localization of Grx1 in vacuoles [18,28] suggests possible export of Grx1 out of cells, further studies were conducted using induced sputum specimens from non-smokers (n = 15), smokers (n = 11) and patients with COPD (n = 24). As expected, there was a prominent Grx1 immunoreactivity in the cells found in induced sputum, as analyzed by immunocytochemistry and Western blot (Fig 6B). However, Grx1 could also be detected from the induced sputum supernatants of healthy controls, smokers, stable COPD and COPD during exacerbation (Fig 6C). The Grx1 protein levels increased in supernatants obtained from patients with acute exacerbations when compared to non-smokers (p = 0.013), and also, to some extent, when compared to smokers (p = 0.051) (Fig 6D). One explanation for the extracellular Grx1 could be the disruption of the plasma membrane, hence the specimens were analyzed for β-actin and manganese superoxide dismutase (an antioxidant enzyme previously found to be expressed in human alveolar macrophages) [29]; the immunoreactivities for both of these antigens were negative.

## Discussion

The present study suggests that Grx1 is a potential redox modulatory protein regulating the homeostasis of glutathionylated proteins and GSH not only in healthy lung but also in cigarette smokers and in COPD patients. In addition, the presence of Grx1 in the extracellular space may play a role in the replenishment of glutathione in the airways, especially during acute exacerbations where the oxidant stress is increased.

One recent microarray study found no change in Grx1 mRNA levels in the bronchial epithelium of healthy smokers compared to non-smokers [30]. In another study on cultured bronchial epithelial cells, Grx was found to be elevated by 10 fold during the first 10 hours of exposure to cigarette smoke [31]. However, to date no human studies are available on the protein levels or the regulation of Grxs in smokers' lung or in COPD patients. It is well known that cigarette smoke can increase GSH levels in the epithelial lining fluid and cause depletion of intracellular free GSH [6,7]. It appears that after initial GSH depletion intracellular GSH levels increase for reasons that are unknown, one mechanism being increased GSH synthesis [4,7,9]. Oxidative stress also causes accumulation of protein-GSH mixed disulfides [32]. Since the intracellular concentration of cysteine-residues in proteins is higher than the concentration of free GSH, the formation of protein-GSH mixed disulfides has the potential to serve as a significant depository of GSH in living cells. Furthermore it has been shown that in an oxidizing environment, a major fraction of GSH is in fact present as mixed disulfides with proteins [33]. Formation of these mixed disulfides between GSH and proteins may serve both a regulatory and an antioxidant function by protecting the enzymes from irreversible oxidation that might lead to enzyme inactivation. Once oxidative stress has been alleviated the protein-GSH mixed disulfides are efficiently reduced by Grxs liberating free glutathione.

**Figure 7:**
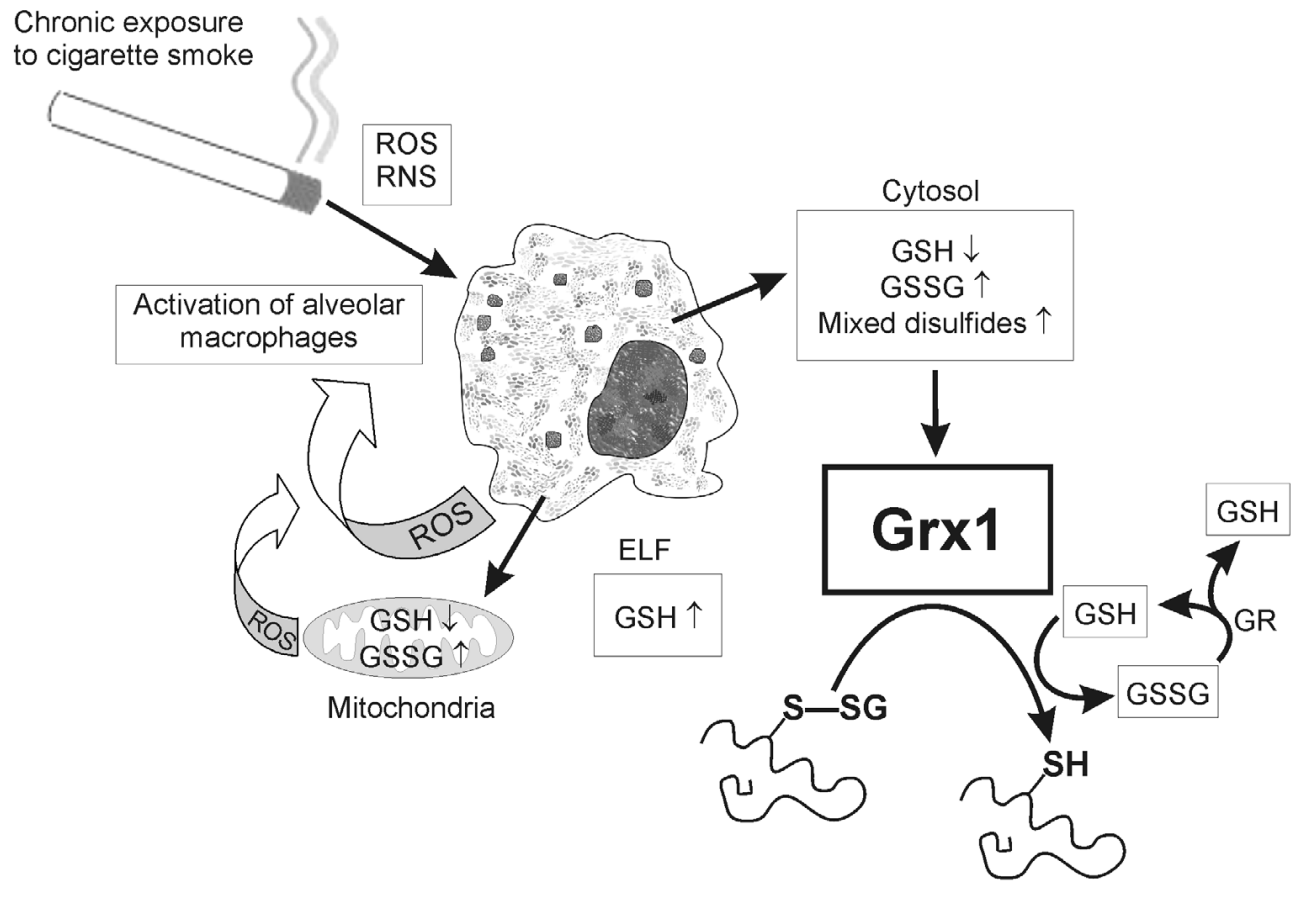
**Suggested role of Grxs in cigarette smoke induced oxidative stress**. The major function of Grx is the reduction of glutathionylated proteins back to their functional states both intracellularly and in the extracellular space with concomitant release of glutathione. ROS, reactive oxygen species; RNS, reactive nitrogen species; GSH, reduced glutathione; GSSG, oxidized glutathione; GR, glutathione reductase; ELF, epithelial lining fluid.

The present study suggests that Grx1 may be involved in the regulation of glutathionylated protein levels and GSH homeostasis in human lung (Fig 7). Immunohistochemical studies showed that Grx1 was mainly expressed in the alveolar macrophages of non-smokers and smokers and at different stages of COPD and that the level of Grx1 decreased according to the severity of the disease. These conclusions are supported by Western blot analysis. Not all macrophages in the sections were Grx1 positive, probably due to presence of various macrophage populations and the age of these cells. Overall, these changes are apparently related both to the total number macrophages present in lung as well as to the differences in the expression levels of Grx in macrophages.

The present study confirmed that Grx1 can be detected from the plasma, as shown previously [19,20], and more importantly demonstrated that Grx1 could also be detected from sputum, both from the cells and supernatants. The latter observation suggests that Grx1 may be exported from alveolar macrophages to the extracellular space (i.e. sputum extract). The levels of Grx1 in the sputum supernatants were significantly higher in acute exacerbations of COPD than in the controls. This suggests that extracellular Grx1 attempts to reduce GSH-mixed disulfides during the oxidative stress in COPD to restore active proteins and to increase the concentration of free glutathione.

The assessment of functional Grx activity from the lung or sputum specimens is difficult since the assays available may not to be specific for Grx alone. In our recent paper [34] we described a new method for measuring the deglutathionylation activity *in vitro*, but also this assay is unsuitable for measuring the glutaredoxin activity in tissue or cell homogenates. In that particular study also other thioredoxin family members showed deglutathionylation activity, further suggesting that the current methods are not specific for the assessment of Grx activity *in vivo*. Supporting the findings in the present study, our recent observations showed that the activity of *E. coli *Grx1, which is similar to human Grx1, is markedly reduced under conditions mimicking oxidative stress [35].

Grx2 expression could not be detected reliably with the methods used here. However, this does not rule out the importance of Grx2 in the antioxidant defense of lung. Grx2 is a mitochondrial protein [14,15] and therefore it might have a role in the regulation of the mitochondrial redox state and apoptotic events. Grx2 clearly needs further investigation in order to better understand the role of thiol oxidoreductases in the antioxidative defense mechanisms in human lung and COPD progression.

The regulation of Grx1 is poorly understood, but recent results of our laboratory and others have suggested minor or non-significant Grx1 induction by oxidants [35,36] and significant Grx1 downregulation by transforming growth factor-beta (TGF-β) [18]. Grx1 is, however, only one of the enzymes that regulate GSH homeostasis in human lung. The rate limiting enzyme in GSH synthesis is GCL and this enzyme has been shown to be transiently induced by oxidative stress, cigarette smoke and COPD [1,8,30,37] but decreased by TGF-β *in vitro *[38] and during COPD progression *in vivo *[9,39]. The decrease of Grx1 in severe COPD/emphysema found here may in fact be associated with a simultaneous downregulation of other GSH associated enzymes in COPD thus further increasing the oxidant burden in the lung. These regulatory pathways clearly need to be studied in more detail.

## Conclusion

In conclusion, Grx1 is a protein closely associated with GSH and reduction of GSH-mixed disulfides. Grx1 is located mainly in alveolar macrophages and the levels are decreased according to severity of COPD. Grx1 can also be detected from sputum supernatants, the levels being higher in acute exacerbations of COPD than in healthy controls. This increase in Grx1 levels may serve as an attempt to restore the functional activity of glutathionylated proteins and to increase the extracellular level of free glutathione. Overall these findings suggest that Grx1 is involved in the regulation of both intracellular and extracellular GSH homeostasis, and associated with the decrease of the GSH dependent antioxidant defense in severe COPD.

## Competing interests

The authors declare that they have no competing interests. The study has not been supported by tobacco industry.

## Authors' contributions

MJP carried out the Western blotting studies, participated in analyzing the immunohistochemical data, performed part of the statistical analysis and drafted the manuscript. PHR participated in selection and collection of patient material, analyzing the immunohistochemical results and performed part of the statistical analysis. THH participated in the design of the study and selection of patient material, and performed part of the statistical analysis. YMS and KMS participated in selection of patient material and analyzing the immunohistochemical results. LWR participated in study coordination and helped to draft the manuscript. VLK conceived the study, and participated in its design and coordination and helped to draft the manuscript. All authors have read and approved the final manuscript.

## Supplementary Material

Additional file 1 Materials and methods. Additional documentation includes a more detailed description of methods used in the study. Click here for file
