# Supplementary material for: Modulation of glutaredoxin in the lung and sputum of cigarette smokers and chronic obstructive pulmonary disease
Source: Respir Res. 2006 Oct 25;7(1):133. doi: 10.1186/1465-9921-7-133 (PMC1633737; doi:10.1186/1465-9921-7-133)
Supplement: Additional file 1 — Materials and methods. Additional documentation includes a more detailed description of methods used in the study. [file 1465-9921-7-133-S1.doc]

**A more detailed description of materials and methods**

**Immunohistochemistry**

The biopsy material was fixed in 10% neutral formalin under vacuum to expand the tissue and to remove air bubbles, or perfused by injecting the fixative with a small-gauge needle and syringe into bronchioles [E1]. After fixing, the specimen was dehydrated and embedded in paraffin. Four micron thick sections were cut from representative paraffin blocks, placed in cold water and sequentially in 40°C water and collected onto 3’-aminopropyltriethylsilane coated glass slides and dried at 37°C o/n. The sections were deparaffinized in xylene, rehydrated through a series of ethanol solutions and finally washed with phosphate buffered saline (PBS). Endogenous peroxidase activity was eliminated by incubation in 1 % H_2_O_2_ in absolute methanol. Washed sections were incubated with 2 % nonfat dry milk in distilled water to diminish background staining and blocked according to instructions of the Vectastain Elite ABC kit (Vector Laboratories Inc, CA, USA). Goat anti-human Grx1 antibody [E2] (1:3000) was used as the primary antibody and biotinylated rabbit anti-goat antibody (Zymed Laboratories Inc, South San Francisco, CA, USA) was used as the secondary antibody (1:300). The avidin-biotin complex was made according to the instructions of the Histostain^TM^ Plus –kit (Zymed) and the duration of aminoethyl carbazole (AEC) (Zymed) color reaction was the same for all samples. The sections were counterstained with Mayer’s hematoxylin (OY Reagena LTD, Kuopio, Finland) and mounted with ImmuMount (Shandon, Pittsburgh, PA, USA). As a negative control, the primary antibody was replaced by PBS or non-immune goat serum (Zymed). The paraffin embedded sections from normal lung and COPD were evaluated independently by two investigators (YS, KS) by grading the staining intensity separately in alveolar macrophages, bronchial epithelial cells and alveolar cells semiquantitatively as follows: 0 = negative, 1 = weak, 2 = moderate, and 3 = intense immunoreactivity.

**Sputum Induction**

Sputum samples were processed immediately after induction according to the ERS task force instructions [E3]. All sputum macroscopically free of salivary contamination was selected and treated with dithioerythritol (DTE, Sigma, Germany) and phosphate-buffered saline. The suspension was centrifuged and the supernatant stored at –80ºC. The pellet was resuspended and the viabilities and absolute numbers of cells were calculated by trypan blue exclusion. Coded cytospins were prepared and stained using the May-Grunwald-Giemsa (MGG) method to obtain cell differential counts. Additional cytospins were frozen in –20ºC for further immunocytochemical assays.

**Western blotting**

Samples containing 40 μg of cellular protein were applied to a 12 % sodium dodecyl sulphate-polyacrylamide gel, separated at 60 mA for 1 hour in 1 x running buffer (25 mM Tris-HCl, pH 8.3, 192 mM glycine and 0.1 % SDS). The proteins were transferred onto Hybond ECL nitrocellulose membrane (Amersham Pharmacia Biotech, UK) at 100 V for 1 hour in 1 x transfer buffer (25 mM Tris, 192 mM glysine and 20 % methanol). The blotted membranes were washed with 1 x TBS-T (20 mM Tris-HCl, pH 7.4, 150 mM NaCl and 0.1% Tween 20), blocked with 10 % nonfat dry milk in 1 x TBS-T, washed again and incubated with antibodies to Grx1 [E2] or Grx2 [E4] (both a gift from A. Holmgren, Karolinska Institut, Stockholm, Sweden) or β-actin (Sigma Aldrich, St. Louis, MO, USA). The incubations were followed by treatment with the appropriate horseradish peroxidase-conjugated secondary antibody (anti-goat for Grx1, anti-rabbit for Grx2 and anti-mouse for β-actin) in 5 % nonfat dry milk in 1 x TBS-T. After washing, the peroxidase was detected using an enhanced chemiluminescense (ECL) system (Amersham) with the luminol excitation imaged on X-ray film (Hyperfilm ECL, Amersham). The intensities of the bands were evaluated densitometrically using Kodak 1D Image Analysis Software (Eastman Kodak Companies, NY, USA)

**References**

E1. Dail DH, Hammar SP: *Pulmonary pathology.* Springer-Verlag, New York 1994, p. 1-17

E2. Nakamura H, Vaage J, Valen G, Padilla CA, Bjornstedt M, Holmgren A: **Measurements of plasma glutaredoxin and thioredoxin in healthy volunteers and during open-heart surgery.** *Free Radic Biol Med* 1998, **24:** 1176-1186

E3. Djukanovic R, Sterk PJ, Fahy JV, Hargreave FE: **Standardised methodology of sputum induction and processing.** *Eur Respir J Suppl* 2002, **37:** S1-S55

E4. Lundberg M, Johansson C, Chandra J, Enoksson M, Jacobsson G, Ljung J, Johansson M, Holmgren A: **Cloning and expression of a novel human glutaredoxin (Grx2) with mitochondrial and nuclear isoforms.** *J Biol Chem* 2001, **276:** 26269-26275
